# Amoxicillin-induced Steven–Johnson syndrome in a 13-year-old female a case report

**DOI:** 10.1097/MS9.0000000000003518

**Published:** 2025-06-23

**Authors:** Mohammedsadeq A. Shweliya, Mohammed Hamed Ibrahium Badi, Yusur Aamer, Hassan H. Eladl, Abdulhadi M. A. Mahgoub

**Affiliations:** a Department of Surgery, University of Baghdad College of Medicine, Baghdad, Iraq; b Department of Medicine, University of Khartoum Faculty of Medicine - EDC Khartoum, Sudan; c Department of Gynaecology, Mustansiriyah University, Baghdad, Iraq; d Faculty of Medicine, Ain Shams University, Cairo, Egypt; e Faculty of Medicine, University of Gezira, Wad-Medani, Gezira, Sudan

**Keywords:** amoxicillin, case report, drug hypersensitivity reactions, SJS, Stevens–Johnson syndrome

## Abstract

**Introduction and importance:**

Stevens–Johnson syndrome (SJS) is a rare but potentially life-threatening mucocutaneous reaction typically triggered by medications. Although antibiotics are among the known causes, amoxicillin is only rarely implicated.

**Case presentation:**

This report highlights a case of amoxicillin-induced SJS in a 13-year-old female of Middle Eastern descent from Iraq, who presented to the emergency department with a 3-day history of exfoliative, painful, burning skin rash accompanied by episodes of bloody vomiting. The patient, with no history of drug allergies, was prescribed amoxicillin for respiratory symptoms. Within 2 days of initiating the medication, she developed a papular rash that rapidly evolved into vesicular lesions with significant mucosal involvement, including painful oral ulcers and cracked lips.

**Clinical discussion:**

After a misdiagnosis of chickenpox led to additional medications, her condition further deteriorated, resulting in an emergency department visit and initiation of supportive care. Over the next 10 days, she received intensive management and gradually improved until discharge.

**Conclusion:**

This case emphasizes the critical need for clinicians to maintain a high index of suspicion for drug-induced SJS, especially in pediatric patients receiving commonly prescribed antibiotics like amoxicillin. Early recognition and prompt discontinuation of the offending drug are crucial to reduce morbidity and improve patient outcomes.

## Introduction

Stevens–Johnson syndrome (SJS) is a rare but severe mucocutaneous reaction characterized by widespread skin lesions and mucosal involvement, often triggered by medications^[^1,2^]^. Although being rare, it is reported that Amoxicillin can induce SJS^[^3^]^. Drug hypersensitivity reactions are an increasing public health concern worldwide, contributing to a significant number of hospital admissions, ranging from 0.1% to 16.8%^[^4^]^. Severe Cutaneous Adverse Reactions (SCARs), such as SJS and Toxic Epidermal Necrolysis (TEN), have a reported incidence of approximately 1.4–6 cases per million person-years^[^5^]^. The condition carries significant morbidity and mortality, with a higher mortality rate observed in younger children^[^6^]^. This case report presents a detailed account of a 13-year-old female who developed SJS following amoxicillin administration for suspected acute tonsillitis, the report will also discuss the challenges in managing SJS, particularly in pediatric patients, as evidenced in other case studies^[^6,7^]^.

**HIGHLIGHTS**

- This report highlights a case of Amoxicillin-induced Stevens–Johnson syndrome (SJS) in a 13-year-old female of Middle Eastern descent from Iraq.
- Emphasizes the critical need for clinicians to maintain a high index of suspicion for drug-induced SJS, especially in pediatric patients receiving commonly prescribed antibiotics like amoxicillin.
- Providing literature review for relevant studies.

### Case presentation

A 13-year-old female initially presented with cough, rhinorrhea, sore throat, and dysphagia. Based on these symptoms, she was prescribed amoxicillin for suspected acute tonsillitis. Two days thereafter, she started developing a papular skin rash, which soon involved her mucocutaneous membranes, leading to painful oral ulcers, cracked lips, and bloody vomiting. Patient misdiagnosed as chickenpox and was treated with ceftriaxone, fluconazole, acyclovir, and ondansetron. The rash first appeared on her hands and arms, and, over 5 days, progressed to involve her face, chest, and lower limbs, evolving from papules to vesicles.

**Figure 1.**
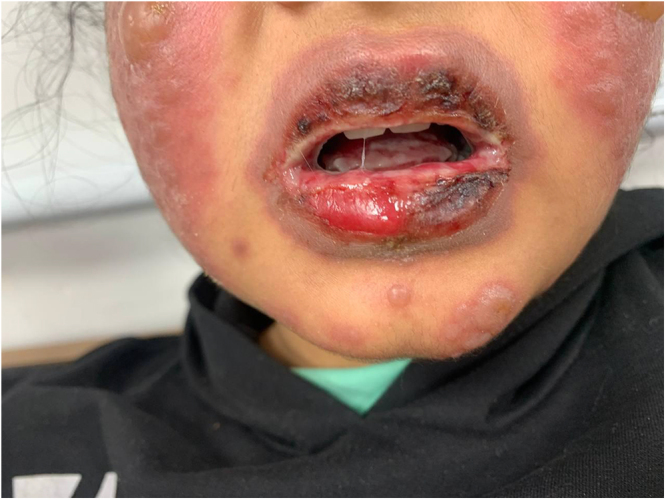
Illustrates the characteristic facial lesions, including mucosal involvement with oral ulceration and cracked lips.

**Figure 2.**
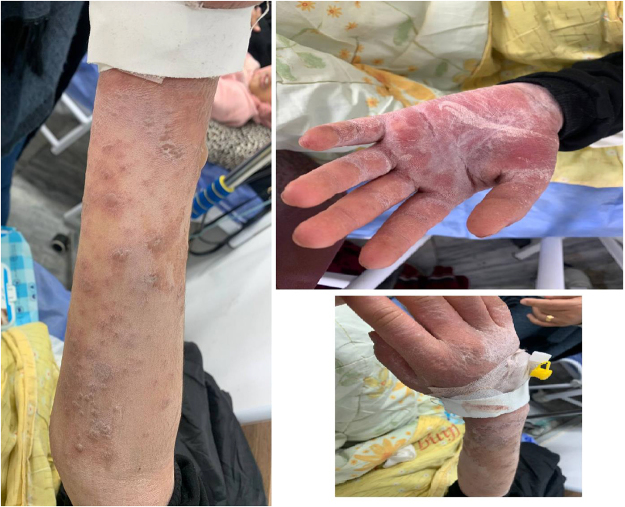
Demonstrates the papular-to-vesicular lesions observed on the patient’s hands, highlighting the progression and severity of the cutaneous reaction.

On examination, the patient was alert but in distress. The findings included red, congested eyes, multiple oral lesions with cracked, edematous lips and ulcers, and blister-like lesions with an erythematous base on both cheeks. A diffuse non-blanching rash was present over her body, with less severe involvement of the lower limbs (Figs. 1 and 2).

Her vital signs were as follows: temperature 37.8°C, respiratory rate 20 breaths per minute, oxygen saturation 99% on room air, blood pressure 120/70 mmHg, and pulse rate 134 beats per minute.

Laboratory investigations showed a white blood cell count of 10.9 × 10^3^/µL, with a high neutrophil percentage (76.7%; normal: 39.3%–73.7%), reduced lymphocyte percentage of 17.6% (normal: 18%–45.3%). Biochemical studies demonstrated an elevated aspartate transaminase level of 36.5 U/L (normal <34 U/L) and a positive C-reactive protein test. Other parameters, including electrolyte levels, urea, creatinine, and additional liver enzymes, remained within normal ranges.

The patient was treated with aggressive supportive care. Intravenous normal saline was administered at a rate of 500 ml per hour. Additionally, she received glucose saline (0.45% sodium chloride with 5% dextrose) every 6 hours, paracetamol for pain management, omeprazole for gastric protection, an antihistamine for symptomatic relief, and topical agents to ease the burning sensation associated with her skin lesions.

She was admitted to hospital and continued on the same management. Her condition improved gradually, and the patient discharged from hospital 10 days later.

### Discussion

This case of amoxicillin-induced SJS in a 13-year-old female underscores the potentially life-threatening nature of drug-induced SCARs and emphasizes the need for early diagnosis and prompt management. SJS is a rare but serious adverse reaction characterized by widespread epidermal detachment and mucosal involvement. In this patient, the rapid progression from a papular rash to vesicular lesions, along with significant mucocutaneous involvement, mirrors the clinical patterns described in the literature. SJS can be misdiagnosed due to its resemblance of other conditions, such as chickenpox. Both diseases may initially present with macules and mucosal involvement. However, the chickenpox macule is typically pruritic while being painful in SJS, with more severe mucosal involvement. Another clinically helpful differentiating feature is that SJS is associated with neutrophilia while chickenpox is associated with lymphocytosis, which may proceed the onset of rash. SJS should be differentiated from other diseases with dermatological involvement. DRESS syndrome which characterized by eosinophilia, drug-induced skin eruption, and a major organ involvement, with liver being the most commonly involved organ. Main culprit antibiotics involved in DRESS syndrome are sulfonamide, nitrofurantoin, vancomycin and minocycline^[^8^]^. Amoxicillin is also a known trigger for morbilform rash, which is characterized by a rash that blanch with pressure, sparing the face and associated with low-grade fever^[^9^]^. Another important differentiation diagnosis is staphylococcal scalded skin syndrome (SSSS). In SSSS, there are vesicles, erosions but no mucous membrane involvement and Nikolsky’s sign is positive^[^10^]^.

While amoxicillin is not commonly associated with SJS, misdiagnosis, and polypharmacy likely exacerbated the condition. This emphasizes the importance of proper and an indication-based prescription of antibiotics in tonsillitis when the underlying cause is viral, and avoids exposing the patient to unnecessary medication. Furthermore, setting clear and appropriate approach to establish early diagnosis may help applying therapeutic strategies early in the disease course and preventing unnecessary intervention^[^11^]^.

Several studies support the association between amoxicillin and SJS/TEN. For instance, Pejcic *et al*^[^12^]^ conducted a systematic review that included 64 patients with amoxicillin-induced SJS/TEN, finding that nearly half of the cases presented as TEN with a significant proportion experiencing prolonged hospital stays and severe complications. Their findings highlight that both amoxicillin alone and in combination with clavulanic acid can precipitate these reactions across a broad age range, reinforcing the need for clinicians to vigilantly monitor for early signs of drug hypersensitivity^[^12^]^.

The distinct safety profile of amoxicillin/clavulanic acid, as highlighted by Salvo *et al*^[^13^]^, demonstrates that combinations may increase the risk of adverse outcomes, including higher rates of SJS and additional complications such as hepatitis. While our patient received amoxicillin alone, these findings suggest that even single-agent therapy can lead to severe outcomes, warranting caution regardless of combination therapy^[^14^]^.

Case reports by Nyamagoud *et al*^[^1^]^ and Srinivasan *et al*^[^2^]^ further corroborate the severity of amoxicillin-induced SJS. In the report by Nyamagoud *et al*^[^1^]^, a 42-year-old male developed SJS after taking amoxicillin for a sore throat, which parallels the mucocutaneous manifestations observed in our patient. Srinivasan *et al*^[^2^]^ also described a case where amoxicillin-induced SJS was confirmed using validated causality assessments, and the report stressed the importance of timely diagnosis and drug cessation to prevent further morbidity^[^1^]^.

The collective evidence from these studies, as summarized in our literature review table (Table 1). Although this case highlights the recognition of SJS in a pediatric patient and provides detailed documentation and a literature review, the findings are based on an individual case report, with no control group present, limiting the generalizability of these findings. In conclusion, this case highlights the critical role of prompt diagnosis and supportive management in amoxicillin-induced SJS. The literature supports that while SJS/TEN remains a rare complication, the potential for rapid progression and high morbidity necessitates early drug withdrawal and comprehensive supportive care. Clinicians should remain vigilant for signs of drug hypersensitivity in patients receiving amoxicillin, ensuring that any early manifestations are swiftly addressed to prevent severe complications.

**Table 1:** Literature review – amoxicillin-induced Stevens–Johnson syndrome (SJS).

Study no.	Authors and year	Study design	Sample size/population	Key findings	Conclusions
1	Pejcic AV *et al* (2023, 8)	Systematic Review	64 patients (age: 1.5–80 years, median 24.5)	−46.9% TEN, 43.8% SJS, 1.6% SJS/TEN overlap.	Amoxicillin (alone or with clavulanic acid) can trigger SJS/TEN across all ages. Clinicians must recognize early signs and discontinue the drug promptly.
				- Hospital stay: 3–70 days (median 16).	
				- High complications, sequelae, and mortality.	
				- SJS/TEN onset varies (immediate to delayed).	
2	Milosavljević MN *et al* (2021, 9)	Narrative Review	36 patients (age: 3–77 years, median 32.5)	−66.7% TEN, 27.8% SJS, 5.6% overlap.	Acetaminophen may cause SJS/TEN. Early drug withdrawal and supportive care are critical. Clinicians should consider acetaminophen as a potential trigger.
				- Symptom onset: median 3 days post-acetaminophen.	
				−77.8% received supportive care; 69.4% corticosteroids.	
				- All survived; 13.9% had sequelae.	
3	Salvo F *et al* (2007, 10)	Retrospective Database Analysis	2183 reports (1095 amoxicillin; 1088 combo)	- Skin reactions: 82% (amoxicillin) vs. 76% (combo).	Amoxicillin/clavulanic acid has a distinct safety profile (higher gastrointestinal/hepatic risks). Inappropriate β-lactam use in Italy necessitates cautious prescribing.
				- Combo linked to higher SJS, hepatitis, and purpura risk.	
				- Hepatitis reporting rate: 9× higher with combo.	
4	Nyamagoud S *et al* (2020, 1)	Case Report	1 patient (42-year-old male)	- SJS developed after amoxicillin was used to treat sore throat.	Amoxicillin-induced SJS is rare but severe. Clinicians must recognize hypersensitivity signs early and discontinue the drug immediately.
				- Presented with widespread rash, fever, and mucosal lesions.	
5	Srinivasan S *et al* (2019, 2)	Case Report	1 patient (63-year-old male)	- Diagnosed via Naranjo (score 7) and WHO causality scales.	Amoxicillin-induced SJS requires prompt diagnosis and drug cessation. Antibiotic misuse in regions like India necessitates strict adherence to prescribing guidelines to prevent severe ADRs like SJS/TEN.
				- Treated with antibiotics (cefotaxime, chloramphenicol) and steroids.	
				- Overuse of antibiotics in developing countries increases SJS/TEN risk.	

ADR, adverse drug reaction; SJS, Stevens–Johnson syndrome; TEN, toxic epidermal necrolysis.

## Data Availability

Data are available from the corresponding or submitting authors upon reasonable request.

## References

[1] Nyamagoud S Deshpande K Kotian A. Amoxicillin induced Steven Johnson’s syndrome: a case report. J. Drug Deliv. Ther 2020;10:220–22.

[2] Srinivasan S Karthikeyan E Sivaneswari S. Clinical condition and medication therapy of amoxicillin-induced Stevens-Johnson syndrome: a case report. Aging Med 2019;2:227. 10.1002/agm2.12088 PMC8445041 34553109

[3] Korkut M Bedel C. A case report of Stevens-Johnson syndrome, a dermatological emergency, secondary to amoxicillin-clavulanic acid use. J Pak Assoc Dermatol 2020;30:519–21.

[4] Pourpak Z Fazlollahi MR Fattahi F. Understanding adverse drug reactions and drug allergies: principles, diagnosis and treatment aspects. Recent Pat Inflamm Allergy Drug Discov 2008;2:24–46. 19075990 10.2174/187221308783399289

[5] Mockenhaupt M. Epidemiology of cutaneous adverse drug reactions. Allergol Select 2017;1:96. 30402608 10.5414/ALX01508E PMC6039997

[6] Zanetti C. Wound care in a child suffering from Stevens-Johnson syndrome in PICU: case report. Inferm J 2024;3:9–13.

[7] Ramassamy S. A narrative review on evaluation of causal drug in cutaneous drug eruptions: challenges, current state, and the path ahead. Int J Adv Med Health Res 2024;11:80–94.

[8] Volpe A Marchetta A Caramaschi P. Hydroxychloroquine-induced DRESS syndrome. Clin Rheumatol 2008;27:537–39. 17952481 10.1007/s10067-007-0772-1

[9] Bachot N Roujeau JC. Differential diagnosis of severe cutaneous drug eruptions. Am J Clin Dermatol 2003;4:561–72. 12862499 10.2165/00128071-200304080-00006

[10] Liy-Wong C Pope E Weinstein M. Staphylococcal scalded skin syndrome: an epidemiological and clinical review of 84 cases. Pediatr Dermatol 2021;38:149–53. 10.1111/pde.14470 33283348

[11] Frantz R Huang S Are A. Stevens–Johnson syndrome and toxic epidermal necrolysis: a review of diagnosis and management. Medicina (B Aires) 2021;57:895. 10.3390/medicina57090895 PMC8472007 34577817

[12] Pejcic AV Milosavljevic MN Folic M. Amoxicillin-associated Stevens-Johnson syndrome or toxic epidermal necrolysis: systematic review. J Chemother 2023;35:75–86. 35285784 10.1080/1120009X.2022.2051128

[13] Salvo F Polimeni G Moretti U. Adverse drug reactions related to amoxicillin alone and in association with clavulanic acid: data from spontaneous reporting in Italy. J Antimicrob Chemother 2007;60:121–26. 17449881 10.1093/jac/dkm111

[14] Milosavljević MN Pejčić AV Milosavljević JZ. A review of published cases of Stevens-Johnson syndrome and toxic epidermal necrolysis associated with the use of acetaminophen. Cutan Ocul Toxicol 2021;40:280–92. 34152866 10.1080/15569527.2021.1942896

